# Central Nervous System Infection with Other Endemic Mycoses: Rare Manifestation of Blastomycosis, Paracoccidioidomycosis, Talaromycosis, and Sporotrichosis

**DOI:** 10.3390/jof5030064

**Published:** 2019-07-18

**Authors:** Carol A. Kauffman

**Affiliations:** Infectious Diseases Section, Veterans Affairs Ann Arbor Healthcare System, University of Michigan Medical School, Ann Arbor, MI 48105, USA; ckauff@umich.edu; Tel.: +734-845-3460; Fax: +734-845-3290

**Keywords:** blastomycosis, paracoccidioidomycosis, talaromycosis, sporotrichosis, endemic mycoses, dimorphic fungi, CNS infection

## Abstract

The central nervous system (CNS) is not a major organ involved with infections caused by the endemic mycoses, with the possible exception of meningitis caused by *Coccidioides* species. When CNS infection does occur, the manifestations vary among the different endemic mycoses; mass-like lesions or diffuse meningeal involvement can occur, and isolated chronic meningitis, as well as widely disseminated acute infection that includes the CNS, are described. This review includes CNS infection caused by *Blastomyces dermatitidis*, *Paracoccidioides brasiliensis*, *Talaromyces marneffei*, and the *Sporothrix* species complex. The latter is not geographically restricted, in contrast to the classic endemic mycoses, but it is similar in that it is a dimorphic fungus. CNS infection with *B. dermatitidis* can present as isolated chronic meningitis or a space-occupying lesion usually in immunocompetent hosts, or as one manifestation of widespread disseminated infection in patients who are immunosuppressed. *P. brasiliensis* more frequently causes mass-like intracerebral lesions than meningitis, and most often CNS disease is part of disseminated infection found primarily in older patients with the chronic form of paracoccidioidomycosis. *T. marneffei* is the least likely of the endemic mycoses to cause CNS infection. Almost all reported cases have been in patients with advanced HIV infection and almost all have had widespread disseminated infection. Sporotrichosis is known to cause isolated chronic meningitis, primarily in immunocompetent individuals who do not have *Sporothrix* involvement of other organs. In contrast, CNS infection in patients with advanced HIV infection occurs as part of widespread disseminated infection.

## 1. Introduction

Central nervous system (CNS) infections caused by endemic mycoses, with the exception of coccidioidal meningitis, are rare. The HIV epidemic led to the increasing recognition of CNS involvement as a component of disseminated infection with several of the endemic mycoses, notably *Histoplasma capsulatum* and *Talaromyces marneffei* (formerly *Penicillium marneffei*), and with *Sporothrix* species, which share the property of being an environmentally-acquired dimorphic fungus. Several of these fungi, notably *Sporothrix* species, *Histoplasma capsulatum*, and *Blastomyces dermatitidis*, have been known for decades to cause isolated chronic meningitis not associated with disseminated infection. Similarly, it has been known that patients with the chronic form of paracoccidioidomycosis can develop parenchymal brain lesions, as well as more common pulmonary, skin, and mucous membrane lesions. However, in all of these endemic mycoses, CNS lesions are uncommon or rare. This review encompasses CNS involvement found in blastomycosis, paracoccidioidomycosis, talaromycosis, and sporotrichosis, with a focus on the clinical and diagnostic aspects of these infections (Table 1, Table 2, Table 3 and Table 4). CNS infections associated with histoplasmosis and coccidioidomycosis are dealt with in separate articles in this issue of the *Journal of Fungi.*

## 2. Blastomycosis (Table 1)

*Blastomyces dermatitidis* is a thermally dimorphic fungus, growing as a mold in the environment; at 37 °C in tissues, the organism converts to the yeast form. Infection develops when conidia are aerosolized from the environment and inhaled [1,2]. A second species, *Blastomyces gilchristii*, is found in the endemic area in North America, but is indistinguishable from *B. dermatitidis* in regard to both growth characteristics and clinical disease. In the last year, another species, *Blastomyces helicus*, has been identified as a cause of severe disseminated infection in a small number of humans and several animal species [3].

### 2.1. Epidemiology

Blastomycosis is seen most frequently in areas of the United States and Canada that border the Great Lakes and the Mississippi and Ohio River valleys. Less commonly, well-documented cases of blastomycosis have been reported from other non-endemic areas in the United States and from Latin America, Africa, and Asia [4,5]. Most cases are sporadic and related to outdoor activities that lead to exposure to the organism, but there are cases in which routine daily activities with no obvious exposure have resulted in infection. Outbreaks have been reported among groups engaged in activities that involve the disruption of soil and vegetation in areas adjacent to waterways [4]. More cases are reported in men than women, presumably related to greater environmental exposure. In some highly endemic areas, dogs are at high risk for blastomycosis, and not infrequently, both dogs and their owners develop infection with *B. dermatitidis* [6]. 

Among immunosuppressed patients, blastomycosis occurs less often than the other endemic mycoses. It has been reported in only a small number of transplant recipients [2,7,8,9,10,11] and in even fewer patients who have received tumor necrosis factor-alpha (TNF) inhibitors [11,12], or had hematological malignancies [7,11]. Patients with HIV/AIDS may be at a higher risk, and have been noted to develop severe infection with a high fatality rate [13]. 

### 2.2. Clinical Manifestations

The lungs are the primary portal of entry of *B. dermatitidis*, and thus, acute and chronic pneumonias are common presentations of blastomycosis. As is true of other endemic mycoses, asymptomatic hematogenous dissemination likely occurs in many patients. With blastomycosis, the typical end organs to which the organism disseminates are skin, bones, and the male genitourinary tract. By the time the patient seeks medical attention for symptoms related to the affected end organ, the initial lung lesions may have totally resolved [14]. 

CNS involvement is not common with blastomycosis, but numerous individual case reports, several small series, and an older large review of cases reported prior to 1978 provide a basis for understanding the clinical characteristics associated with CNS infection [7,11,13,15,16,17]. CNS blastomycosis takes two major forms: intracranial space-occupying lesions and meningitis. Some patients have meningitis as well as parenchymal lesions. Isolated CNS infection can occur without involvement of other sites [7,11,15,16,17]; however, more commonly, CNS infection is part of widespread disseminated blastomycosis [7,11,15,17]. CNS involvement is more common in immunocompromised patients [13], but in some reports, most patients had no underlying illnesses [17]. 

Patients who present with intracranial mass lesions are usually thought to have a brain tumor or bacterial abscess. Clinical findings include a headache, which is the predominant symptom, focal neurological signs, mental status changes, seizures, and signs of increased intracranial pressure. Either single or multiple intracranial lesions may be present. Imaging studies are crucial for diagnosis.

Meningitis is more common than mass lesions. When isolated meningitis occurs, without manifestations of infection elsewhere, the course is generally subacute, with an increasing headache, mental status changes, and sometimes signs of increasing intracranial pressure. In patients who have disseminated blastomycosis, the neurological symptoms may be overlooked in the patient who has severe pulmonary manifestations, for example, and CNS involvement is only found when imaging studies are performed.

### 2.3. Imaging

Magnetic resonance imaging (MRI) is the most sensitive imaging technique that can be used to detect mass lesions due to blastomycosis. Single or multiple ring-enhancing lesions are typical, and any area of the brain can be involved. If meningitis is present, meningeal enhancement can be seen. 

### 2.4. Diagnosis

Growth of *B. dermatitidis* in the laboratory is the definitive diagnostic test. Because the organism grows slowly, histopathological examination with silver or periodic acid Schiff (PAS) stains of tissue obtained from an involved site can give a rapid presumptive diagnosis and allow earlier initiation of antifungal therapy. The yeast form of *B. dermatitidis* has a distinctive appearance: the organisms are 8–20 μm in diameter; have a thick, double-contoured, refractile cell wall; and produce a single broad-based bud, which often remains attached until it is almost the size of the mother cell [1]. 

Cerebrospinal fluid (CSF) findings in patients with meningitis show lymphocytic pleocytosis, with counts ranging from 30 to 3000 cell/μL [11,15,17]; uncommonly, a predominance of neutrophils can be seen [11,18]. Protein is elevated and can be as high as 300–400 mg/dL [11,19]. Glucose is generally moderately decreased to between 30 and 40 mg/dL, but has been reported to be as low as 11 mg/dL [20]. *B. dermatitidis* can be grown from the CSF of patients who have meningitis, but not as frequently as one would hope [11,15]. 

CSF results are most helpful for patients who have meningitis. Lumbar puncture is not encouraged as a diagnostic test for patients who have mass lesions because of the risks involved, and in addition, the diagnostic yield is low. Patients who have isolated intracerebral mass lesions have the diagnosis made only after biopsy of the lesion. When a patient who has documented disseminated blastomycosis has neurological findings and abnormal neuroimaging, a diagnosis of CNS blastomycosis can be made without a brain biopsy. 

A presumptive diagnosis can be made while awaiting culture results by detection of the *B. dermatitidis* antigen using an enzyme immunoassay for serum or urine [21,22]. Cross reactivity with *Histoplasma capsulatum* commonly occurs because the two organisms share similar galactomannans in their cell walls. Antigen detection in the CSF can be extremely useful in cases of CNS blastomycosis [11]. In contrast to several other endemic mycoses, antibody detection of *B. dermatitidis* in the CSF has not proven useful. Whether a more recently developed antibody assay could prove useful in CSF for diagnosis remains to be seen [23].

### 2.5. Treatment

When *B. dermatitidis* involves the CNS, initial treatment should involve a lipid formulation of amphotericin B, preferably liposomal amphotericin B, 5 mg/kg daily for 4–6 weeks [24]. Following this, an oral azole agent should be given for at least 12 months [24]. Immunosuppressed patients or those who have had relapsing CNS infection often require life-long therapy [11,25,26]. The Infectious Diseases Society of America (IDSA) Guidelines recommend itraconazole or fluconazole as the oral azole agent [24]. Both have been reported to be effective in small numbers of patients [11,17,27,28,29]. 

An increasing number of reports show that voriconazole, which is more active than fluconazole against *B*. *dermatitidis* and which achieves higher levels in the CNS than itraconazole, is effective for treating CNS blastomycosis [11,20,26,29,30,31,32,33,34,35]. The dose that should be used is 400 mg twice daily for the first day, followed by 200–400 mg twice daily. Because of high inter-patient variability in serum concentrations of voriconazole, it is essential to measure serum trough levels, aiming for concentrations of 2–5 μg/mL in order to achieve reasonable concentrations within the CNS. Overall, outcomes for severe blastomycosis, including CNS infection, have improved with the advent of effective oral azole antifungal agents [2]. 

## 3. Paracoccidioidomycosis (Table 2)

*Paracoccidioides brasiliensis* and, less commonly, the more recently described *Paracoccidioides lutzii,* cause paracoccidioidomycosis. These dimorphic fungi are found in the soil in the mold form that produces conidia, which cause infection when inhaled. In tissues and at 37 °C, the mold converts to the yeast form. Infection may remain localized to the entry site, the lungs, or may result in dissemination to many different organs.

### 3.1. Epidemiology

Paracoccidioidomycosis only occurs in Latin America. It is most commonly seen in several South American countries, notably Brazil, but also Argentina, Venezuela, Ecuador, and Colombia [36]. Infection occurs more than ten times more frequently in men than women, and most often occurs in middle-aged to older men [37]. The sexual difference is likely partly related to environmental exposure, but possibly also to the inhibitory effects of estrogens on growth of the organism [37]. For most patients, it is likely that infection occurs early in life, but disease manifestations occur decades later, presumably with the reactivation of latent infection. 

There are increasing reports of paracoccidioidomycosis in patients who have HIV infection [38,39] and a few case reports of paracoccidioidomycosis in patients who have received a solid organ transplant or immunosuppressive therapy with biological agents [39,40,41,42]. CNS involvement does not appear to be more frequent in patients who have HIV infection or immunosuppressive conditions [38,39]. 

### 3.2. Clinical Manifestations

The clinical manifestations of paracoccidioidomycosis are usually separated into two characteristic forms. The chronic form accounts for about 90% of cases, is seen mostly in older men, and shows slow progression over the years, primarily involving the lungs, the skin, and mucous membranes of the head and neck. The acute/subacute or juvenile form is present in about 10% of cases, occurs mostly in children and young adults, and manifests widespread dissemination to the liver, spleen, lymph nodes, and skin. There can be overlap with these two types of disease, and immunosuppressed patients usually manifest rapidly progressive disease involving the lungs, liver, spleen, and skin [39].

Involvement of the CNS has been noted to occur in 10–15% of cases of paracoccidioidomycosis and is found most often in the chronic form of the disease [37,43,44]. Almost all patients have concurrent evidence of disseminated infection, although in some cases, neurological symptoms are the first manifestation of disseminated paracoccidioidomycosis. Rarely, isolated CNS infection has been reported [45]. 

Clinical manifestations of CNS paracoccidioidomycosis are primarily those associated with space-occupying lesions, rather than meningitis; headache, seizures, focal weakness, and gait disturbance with cerebellar involvement are seen. Signs of increased intracranial pressure may be found; few patients have overt signs of meningeal involvement. 

### 3.3. Imaging

Imaging using CT or MRI plays an important role in defining CNS involvement [43,45,46]. Single or multiple space-occupying lesions are the most common finding; most are seen in the cerebrum, but involvement of the cerebellum and spinal cord also occur. When contrast material is administered, ring-like enhancement can be seen, and surrounding tissue shows varying amounts of edema [46]. In some cases, meningeal enhancement, which is greatest near the affected region, can be seen, as can hydrocephalus. 

### 3.4. Diagnosis

The diagnosis of paracoccidioidomycosis is established by the growth of *P. brasiliensis* in culture, but because the yeast form is quite distinctive, early identification can be established from tissue biopsies stained with silver or PAS stains or from secretions treated with calcofluor white. The organism appears as a thick-walled yeast with multiple narrow-based buds attached around the cell wall. However, the diagnosis of CNS paracoccidioidomycosis is difficult because the organism is rarely seen or grown from the CSF. 

Few reports comment on CSF findings, most likely because CNS paracoccidioidomycosis primarily involves the parenchyma rather than the meninges and lumbar puncture is often contraindicated. Findings of moderately increased protein, normal glucose, and few mononuclear cells provide the clinician with little help [43].

Most times, the diagnosis of CNS paracoccidioidomycosis is established presumptively when a patient who has documented disseminated paracoccidioidomycosis has symptoms and signs of CNS involvement and abnormal neuroimaging findings. Biopsy of a CNS lesion will confirm the diagnosis, but is rarely needed; the histopathology is that of a granulomatous response with necrosis.

Serum antibody tests using techniques that include immunodiffusion, counter- immunoelectrophoresis, and an enzyme immunoassay are available in endemic areas, but their sensitivity and specificity vary greatly, and none have proved useful for testing CSF [47]. On the other hand, at least one group has used an antigen assay in which Gp43, an extracellular antigen from *P. brasiliensis*, was detected in the CSF of patients with CNS paracoccidioidomycosis [48]. There is cross-reactivity with the enzyme immunoassay for the *Histoplasma capsulatum* antigen (MiraVista Laboratories, Indianapolis, IN) that is performed in serum and urine [49].

### 3.5. Treatment

Two major treatment options are available for the treatment of CNS paracoccidioidomycosis. The drug of choice recommended in the Brazilian guidelines for the clinical management of paracoccidioidomycosis is itraconazole, 200 mg daily for 6 to 12 months [50]. Voriconazole has been shown to be as effective as itraconazole in a randomized open-label pilot study, and for CNS paracoccidioidomycosis, this agent is attractive because it attains higher CNS concentrations than itraconazole [51]. The other option for treating CNS paracoccidioidomycosis is trimethoprim-sulfamethoxazole [50]. Many reports of CNS paracoccidioidomycosis note that this agent or another sulfa formulation were used [43,44,45]. For patients with severe paracoccidioidomycosis, initial therapy with amphotericin B is warranted with step-down to oral itraconazole or voriconazole or to trimethoprim-sulfamethoxazole. The length of treatment that is required for CNS paracoccidioidomycosis is not known, but long-term suppression with trimethoprim-sulfamethoxazole seems reasonable, especially in immunosuppressed patients. The outcome depends on the extent of dissemination and the host’s immune response against the organism. 

## 4. Talaromycosis (Table 3)

*Talaromyces marneffei,* known as *Penicillium marneffei* until a few years ago, is a dimorphic fungus that grows at 25 °C as a mold that produces a distinctive soluble red pigment that diffuses into the agar. When incubated on the appropriate media at 37 °C and in tissues, the organism assumes the yeast form. 

### 4.1. Epidemiology

In the environment, the organism resides in the soil, primarily in southern China and Southeast Asia, and has a close relationship with bamboo rats in the endemic area. The organism has been cultured from various organs, especially the liver, spleen, and lungs, in several different species of bamboo rats and from their burrows. Although infection has been noted in these rats, most animals show no signs of illness. Most patients have no known direct exposure to these rats [52].

Infection with *T. marneffei* was uncommonly reported prior to the AIDS epidemic, but has been subsequently described in several large series of cases from Thailand and Viet Nam [53,54,55,56]. Other forms of immunosuppression, including that associated with TNF-alpha inhibitor therapy, other biological immunotherapy, and organ transplantation, also predispose patients to disseminated talaromycosis [57,58]; much less commonly, immunocompetent persons may develop disseminated infection [59,60].

### 4.2. Clinical Manifestations

Human infection occurs by the inhalation of arthroconidia that form in the mold phase. Similar to histoplasmosis, after conversion to the yeast phase in the lungs, organisms disseminate widely by the hematogenous route, primarily to organs of the reticuloendothelial system. Presumably, many people exposed to the organisms in the environment are infected but able to handle the organism because they have an intact cell-mediated immunity. They have few symptoms and have only a very mild infection that goes undiagnosed. On the other hand, patients who have T cell dysfunction, especially those with advanced HIV/AIDS, cannot contain the organism and manifest widespread disease. Patients with disseminated infection present with fevers, malaise, anorexia, and weight loss and have involvement of the lungs, liver, spleen, lymph nodes, and skin. The skin lesions are usually numerous and appear as umbilicated or ulcerated lesions often involving the face. 

Of all the endemic mycoses, talaromycosis is the least often reported to cause CNS infection. In reviews of disseminated talaromycosis among patients with and without HIV/AIDS and in one paper focused on CNS disease among HIV/AIDS patients, no patients with CNS talaromycosis were described [54,56,61]. Several individual cases of CNS infection, including one in an immunocompetent patient, have been reported [58,59], but the most well-defined series of cases of CNS infection with *T. marneffei* is in HIV/AIDS patients from Viet Nam [53]. Of 677 cases of talaromycosis in patients with HIV/AIDS, 21 (3%) had CNS involvement. All had the acute onset of confusion, agitation, or obtundation, and very few had any symptoms or signs of increased intracranial pressure or meningitis. All had disseminated talaromycosis involving multiple different organs. 

### 4.3. Diagnosis

The diagnosis of talaromycosis is established by growing the organism from blood or tissue biopsy specimens. A presumptive diagnosis can be made quickly by histopathological examination of skin lesions or other biopsy material. The yeasts are 2 μm × 6 μm, elliptical in shape, and divide by fission, rather than budding, as noted for most yeasts; division by fission results in a distinctive septum in the middle of the yeast cell. In the few cases reported, the diagnosis of CNS talaromycosis has been established by growth of the organism from the CSF. [53]. CSF findings are similar to those in cryptococcal meningitis in patients with advanced AIDS in that few cells are present, the protein is normal or only mildly elevated, and glucose is normal or only mildly decreased. 

Serological methods are not well-defined for talaromycosis and have not been reported for patients with CNS infection. It should be noted that cross-reactivity with the enzyme immunoassay for the *H. capsulatum* antigen (MiraVista Laboratories) commonly occurs, and cross-reactivity with the Platelia^®^ *Aspergillus* galactomannan assay has also been reported [49,62]. Antibody assays have not been reported in cases of CNS talaromycosis. 

### 4.4. Treatment

The treatment of CNS talaromycosis, in general, is the same as that of disseminated disease [63]; the only difference with treating CNS disease is that amphotericin B, preferably a lipid formulation, should be given for a longer period of time and at a higher dosage, similar to the situation with histoplasmosis. A dosage of liposomal amphotericin B of 5 mg/kg for 4–6 weeks, followed by step-down therapy with oral itraconazole 200 mg twice daily or higher to achieve trough serum concentrations above 2 μg/mL, is suggested. It seems reasonable that voriconazole could be used rather than itraconazole because of its better penetration into the CNS, but there are no clinical reports to verify this. It must be noted that experience in treating CNS infection is extremely limited. Of the 21 patients reported by Le et al, only three (14%) survived, and these three patients were aggressively treated with amphotericin B within 24 hours of the onset of neurological symptoms [53]. 

## 5. Sporotrichosis (Table 4)

Sporotrichosis is dissimilar from other endemic mycoses in that it is not geographically restricted, but it does share dimorphic growth characteristics with other endemic mycoses. The environmental form is a mold, which assumes the yeast form in tissues. *Sporothrix schenckii* is not a single species, but rather a complex of at least six phylogenetically different organisms, the most important of which are *S. schenckii* and *S. brasiliensis* [64].

### 5.1. Epidemiology

*Sporothrix* species are found in sphagnum moss, decaying wood and plants, and soil [65]. Almost all infections are associated with an inoculation of the organism through the skin when a person is engaged in activities, such as landscaping, gardening, or farming, or experiences trauma. Rarely, the organism can be inhaled into the lungs. Unique to this infection is zoonotic transmission from cats [65,66]. A large outbreak occurring predominantly in women and children and related to infected cats has been ongoing for more than a decade in Brazil. This is centered in Rio de Janeiro, but has also been noted in Sao Paulo and other areas in southern Brazil. The organism in all of these cases has been identified as *S. brasiliensis* [67,68]. 

Early in the HIV epidemic, sporadic cases of sporotrichosis among persons with advanced HIV infection were reported [69,70]. More recently, larger numbers of cases have been reported from South America and other areas [71,72,73,74]. A few cases of infection in patients treated with immunosuppressive drugs have been described [75,76,77]. 

### 5.2. Clinical Manifestations

The classic and well-known clinical picture of sporotrichosis involves a primary nodular lesion that then ulcerates at the site of inoculation, followed by the appearance of subsequent similar lesions along the proximal lymphatic distribution [78]. A single fixed cutaneous lesion that ulcerates can occur, but is less common. Disseminated sporotrichosis is rare and is almost always related to immune suppression, especially advanced HIV infection. The illness can be manifested as multiple cutaneous lesions only or as widespread visceral involvement along with cutaneous lesions [71,72,73,74,79]. The skin lesions tend to be ulcerated and painful and spread diffusely over the trunk, face, and extremities. 

CNS sporotrichosis can present as either isolated chronic meningitis or as part of widespread dissemination. Early reports from the mid-20th century focused on isolated chronic meningitis, and emphasized the extreme chronicity of the infection, the similarity to tuberculous meningitis, the difficulty in establishing a diagnosis, and the poor outcomes [80,81]. This form of meningitis remains rare, but does occur, often in non-immunocompromised hosts [82]. Later reports of patients who had HIV infection were almost always manifested by CNS involvement being but one manifestation of widespread disseminated infection [79,83,84,85]. 

Chronic meningitis usually presents with weeks to months of gradually increasing headaches, ataxia, and confusion. Fever may or may not be present. Over time, signs of increased intracranial pressure may occur. Almost always, the diagnosis is not established for months. Meningitis associated with disseminated infection is manifested by a more acute onset of headaches, confusion, and occasionally seizures or focal findings. 

### 5.3. Diagnosis

The diagnosis of CNS sporotrichosis can be established by growth of the organism in culture. It is uncommon to grow *Sporothrix* species in CSF from patients with isolated chronic meningitis, but the organism will frequently grow from CSF of patients who have AIDS and disseminated sporotrichosis, presumably because there is a far greater burden of organisms in disseminated sporotrichosis [73,74]. The diagnosis of sporotrichosis can be made sooner when CNS involvement is part of disseminated infection; in those cases, biopsy of skin lesions that are always present establishes the diagnosis quickly. In patients who are immunosuppressed, typically large numbers of budding oval to cigar-shaped yeasts can be seen in tissues stained with methenamine silver stain. 

CSF findings vary, depending on the chronicity of the infection and the state of immunosuppression of the host, but generally show an elevated protein level, a decreased glucose level, and an increase in white blood cells [79,80,81,82,83,84,85]. Protein values range from 100 to 500 mg/dL and glucose values range from 10 to 50 mg/dL. The white blood cell response is almost always mononuclear, and ranges between 40 and 500 cells/μL, with a differential showing 70–90% mononuclear cells. Rarely, a predominance of neutrophils can be seen [79].

The role of serology in the diagnosis of sporotrichosis is debated. Latex agglutination and immunodiffusion were explored decades ago; several tests appeared to be sensitive and specific enough to aid in the diagnosis of sporotrichosis, but were never developed commercially [86,87]. A variety of different laboratory-specific enzyme immunoassays exist, which appear to be used mostly within the country of origin [88]. The diagnosis of isolated chronic *Sporothrix* meningitis was the impetus for the development of some of the earlier latex agglutination tests [86], and similar tests have been used in recent years for CNS sporotrichosis in which growth of the organism from CSF is uncommon [82]. 

### 5.4. Treatment

Initial treatment of CNS sporotrichosis should involve a lipid formulation, preferably liposomal amphotericin B, 5 mg/kg daily for 4–6 weeks [89]. This should be followed by oral therapy with itraconazole, 200 mg twice daily for a total of 12 months of therapy. For patients with HIV infection that has not responded to antiretroviral therapy, for patients who have had relapsing infection, and for patients who remain on immunosuppressive drugs, consideration should be given to continuing maintenance azole therapy for life. 

Itraconazole penetrates the CNS poorly, and some patients have been treated with other azoles. However, the response of sporotrichosis to both fluconazole and voriconazole is poor, and these agents are not recommended [89]. Posaconazole has some activity against *Sporothrix* species, and could be an alternative agent if itraconazole is not tolerated [90]; it has been used successfully in an immunosuppressed patient who had sporotrichosis without CNS involvement [75]. Although not recommended, at least one patient who had chronic *Sporothrix* meningitis and who refused treatment with amphotericin B was treated with only itraconazole and had a good response [82]. The overall prognosis of *Sporothrix* meningitis is dependent on the host’s immune response; disseminated infection with CNS involvement in patients with HIV infection is associated with the worst prognosis. 

## Figures and Tables

**Table 1:** Salient Points Regarding Central Nervous System (CNS) Blastomycosis.

**Clinical Aspects**
**Two major manifestations** ➢ Intracranial space-occupying lesions that mimic a brain tumor or abscess and that can occur as an isolated process or with disseminated blastomycosis ➢ Meningitis that is chronic and without other organ involvement causing a headache, mental status changes, and symptoms of increased intracranial pressure OR meningitis that is one manifestation of disseminated infection and that is seen more often in immunosuppressed patients
**Diagnosis**
➢ Definite CNS blastomycosis: positive culture from cerebrospinal fluid (CSF) OR positive culture or histopathology on tissue obtained by brain biopsy ➢ Presumptive CNS blastomycosis: positive culture from another involved site OR positive histopathology from another involved site in a patient with disseminated blastomycosis and symptoms, signs, and radiological findings of CNS disease OR a positive Blastomyces antigen in CSF
**Treatment and Outcome**
➢ Amphotericin B, preferably lipid formulation, 5 mg/kg daily for 4–6 weeks ➢ Step-down therapy with itraconazole, 200 mg twice daily for at least 12 months ➢ Voriconazole, 200–400 mg twice daily preferred for step-down therapy by many ➢ Posaconazole, 300 mg daily, might be efficacious for step-down therapy if above agents are not tolerated ➢ Outcomes generally good with effective antifungal therapy; may have CNS sequelae with mass lesions

**Table 2:** Salient Points Regarding Central Nervous System (CNS) Paracoccidioidomycosis.

**Clinical Aspects**
**Two major manifestations** ➢ Intracranial space-occupying lesions that mimic a brain tumor or abscess that usually occur as one manifestation of disseminated infection in patients with the chronic form of paracoccidioidomycosis ➢ Meningitis is less common; usually occurs as one manifestation of disseminated infection in either an acute or chronic form of paracoccidioidomycosis
**Diagnosis**
➢ Definite CNS paracoccidioidomycosis: positive culture from cerebrospinal fluid (CSF) (uncommon) OR positive culture or histopathology on tissue obtained by brain biopsy ➢ Presumptive CNS paracoccidioidomycosis: positive culture from another involved site OR positive histopathology from another involved site in a patient with disseminated paracoccidioidomycosis and symptoms, signs, and radiological findings of CNS disease ➢ Serology on CSF may be useful for patients with meningitis; not standardized; may be available in reference laboratories in endemic areas
**Treatment and Outcomes**
➢ Amphotericin B, preferably lipid formulation, 5 mg/kg daily for 4–6 weeks ➢ Step-down therapy with itraconazole, 200 mg twice daily for at least 12 months ➢ Voriconazole, 200–400 mg twice daily, might be efficacious for step-down therapy, but little experience ➢ Trimethoprim/sulfamethoxazole (TMP/SMX), 240 mg TMP/1200 mg SMX (or higher) daily for step-down therapy for at least 12 months ➢ Life-long maintenance azole or TMP/SMX therapy may be required ➢ Outcomes depend on CNS damage from mass lesions; sequelae are common

**Table 3:** Salient Points Regarding Central Nervous System (CNS) Talaromycosis.

**Clinical Aspects**
➢ Rare manifestation of talaromycosis ➢ Almost all cases reported in patients with HIV/AIDS with CD4 cells <100/μL ➢ Almost all patients have widespread disseminated infection
**Diagnosis**
➢ Definite CNS talaromycosis: positive culture from cerebrospinal fluid (CSF) ➢ Presumptive CNS talaromycosis: positive culture from blood or another involved site OR positive histopathology from another involved site in a patient with disseminated talaromycosis and symptoms and signs of meningitis ➢ Serology not useful
**Treatment and Outcomes**
➢ Amphotericin B, preferably lipid formulation, 5 mg/kg daily for 4–6 weeks ➢ Step-down therapy with itraconazole, 200 mg twice daily for at least 12 months ➢ Voriconazole, 200–400 mg twice daily, might be efficacious, if itraconazole is not tolerated ➢ Antiretroviral therapy for patients with HIV infection ➢ Outcomes are dismal; most patients have died

**Table 4:** Salient Points Regarding Central Nervous System (CNS) Sporotrichosis.

**Clinical Aspects**
**Two major manifestations** ➢ Isolated chronic meningitis in immunocompetent patients who have symptoms (headache, ataxia, confusion, etc.) for weeks to months ➢ Acute meningitis (headache, mental status changes, seizures) seen almost entirely in patients with HIV/AIDS with CD4 cells <100 μL as one manifestation of disseminated sporotrichosis. Most patients have many cutaneous lesions
**Diagnosis**
➢ Definite CNS sporotrichosis: positive culture from cerebrospinal fluid (CSF) (uncommon in chronic meningitis) ➢ Presumptive CNS sporotrichosis: positive culture from another involved site (generally skin lesion) OR positive histopathology from another involved site in a patient with disseminated sporotrichosis and symptoms and signs of meningitis ➢ Serology on CSF may be useful for chronic meningitis; not standardized; available in reference laboratories
**Treatment and Outcomes**
➢ Amphotericin B, preferably lipid formulation, 5 mg/kg daily for 4–6 weeks ➢ Step-down therapy with itraconazole, 200 mg twice daily for at least 12 months ➢ Posaconazole, 300 mg daily, might be efficacious if itraconazole is not tolerated ➢ Life-long maintenance azole therapy often required ➢ Antiretroviral therapy for patients with HIV infection ➢ Outcomes are poor for patients with HIV infection and disseminated sporotrichosis; have improved with azole therapy for patients with chronic meningitis

## References

[1] Saccente M., Woods G.L. (2010). Clinical and laboratory update on blastomycosis. Clin. Microbiol. Rev..

[2] Smith J.A., Gauthier G. (2015). New developments in blastomycosis. Sem. Respir. Crit. Care Med..

[3] Schwartz I.S., Wiederhold N.P., Hanson K.E., Patterson T.F., Sigler L. (2018). *Blastomyces helicus,* a new dimorphic fungus causing fatal pulmonary and systemic disease in humans and animals in western Canada and the United States. Clin. Infect. Dis..

[4] Castillo C.G., Kauffman C.A., Miceli M.H. (2016). Blastomycosis. Infect. Dis. Clin. N. Am..

[5] Benedict K., Thompson G.R., Deresinski S., Chiller T. (2015). Mycotic infections acquired outside areas of known endemicity, United States. Emerg. Infect. Dis..

[6] Anderson J.L., Dieckman J.L., Reed K.D., Meece J.K. (2014). Canine blastomycosis in Wisconsin: A survey of small-animal veterinary practices. Med. Mycol..

[7] Pappas P.G., Threlkeld M.G., Bedsole G.D., Cleveland K.O., Gelfand M.S., Dismukes W.E. (1993). Blastomycosis in immunocompromised patients. Medicine (Baltimore).

[8] Kauffman C.A., Freifeld A.G., Andes D.R., Baddley J.W., Herwaldt L., Walker R.C., Alexander B.D., Anaissie E.J., Benedict K., Ito J.I. (2014). Endemic fungal infections in solid organ and hematopoietic cell transplant recipients enrolled in the Transplant-Associated Infection Surveillance Network (TRANSNET). Transpl. Infect. Dis..

[9] Gauthier G.M., Safdar N., Klein B.S., Andes D.R. (2007). Blastomycosis in solid organ transplant recipients. Transpl. Infect. Dis..

[10] Grim S.A., Proia L., Miller R., Alhyraba M., Costas-Chavarri A., Oberholzer J., Clark N.M. (2012). A multicenter study of histoplasmosis and blastomycosis after solid organ transplantation. Transpl. Infect. Dis..

[11] Bariola J.R., Perry P., Pappas P.G., Proia L., Shealey W., Wright P.W., Sizemore J.M., Robinson M., Bradsher R.W. (2010). Blastomycosis of the central nervous system: A multicenter review of diagnosis and treatment in the modern era. Clin. Infect. Dis..

[12] Smith J.A., Kauffman C.A. (2009). Endemic fungal infections in patients receiving tumor necrosis factor-α inhibitor therapy. Drugs.

[13] Pappas P.G., Pottage J.C., Powderly W.G., Fraser V.J., Stratton C.W., McKenzie S., Tapper M.L., Chmel H., Bonebrake F.C., Blum R. (1992). Blastomycosis in patients with the acquired immunodeficiency syndrome. Ann. Intern. Med..

[14] Smith J.A., Kauffman C.A. (2010). Blastomycosis. Proc. Am. Thorac. Soc..

[15] Gonyea E.F. (1978). The spectrum of primary blastomycotic meningitis: A review of central nervous system blastomycosis. Ann. Neurol..

[16] Roos K.L., Bryan J.P., Maggio W.W., Jane J.A., Scheld W.M. (1987). Intracranial blastomycoma. Medicine (Baltimore).

[17] Bush J.W., Wuerz T., Embil J.M., Del Bigio M.R., McDonald P.J., Krawitz S. (2013). Outcomes of persons with blastomycosis involving the central nervous system. Diagn. Microbiol. Infect. Dis..

[18] Harley W.B., Lomis M., Haas D.W. (1994). Marked polymorphonuclear pleocytosis due to blastomycotic meningitis: Case report and review. Clin. Infect. Dis..

[19] Friedman J.A., Wijdicks E.F.M., Fulgham J.R., Wright A.J. (2000). Meningoencephalitis due to *Blastomyces dermatitidis*: Case report and literature review. Mayo Clin. Proc..

[20] Lentnek A.L., Lentnek I.A. (2006). Successful management of *Blastomyces dermatitidis* meningitis. Infect. Med..

[21] Connolly P., Hage C.A., Bariola J.R., Bensadoun E., Rodgers M., Bradsher R.W., Wheat L.J. (2012). *Blastomyces dermatitidis* antigen detection by quantitative enzyme immunoassay. Clin. Vaccine Immunol..

[22] Bariola J.R., Hage C.A., Durkin M., Bensadoun E., Gubbins P.O., Wheat L.J., Bradsher R.W. (2011). Detection of *Blastomyces dermatitidis* antigen in patients with newly diagnosed blastomycosis. Diagn. Microbiol. Infect. Dis..

[23] Richer S.M., Smedema M.L., Durkin M.M., Brandhorst T.T., Hage C.A., Connolly P.A., Leland D.S., Davis T.E., Klein B.S., Wheat L.J. (2014). Development of a highly sensitive and specific blastomycosis antibody enzyme immunoassay using *Blastomyces dermatitidis* surface protein BAD-1. Clin. Vaccine Immunol..

[24] Chapman S.W., Dismukes W.E., Proia L.A., Bradsher R.W., Pappas P.G., Threlkeld M.G., Kauffman C.A. (2008). Infectious Diseases Society of America. Clinical practice guidelines for the management of blastomycosis: 2008 update by the Infectious Diseases Society of America. Clin. Infect. Dis..

[25] Chowfin A., Tight R., Mitchell S. (2000). Recurrent blastomycosis of the central nervous system: Case report and review. Clin. Infect. Dis..

[26] Panicker J., Walsh T., Kamani N. (2006). Recurrent central nervous system blastomycosis in an immunocompetent child treated successfully with sequential liposomal amphotericin B and voriconazole. Pediatr. Infect. Dis. J..

[27] Pearson G.J., Chin T.W., Fong I.W. (1992). Case report: Treatment of blastomycosis with fluconazole. Am. J. Med. Sci..

[28] Brick K.E., Agger W.A. (2012). Successful treatment of brainstem blastomycosis with fluconazole. Clin. Med. Res..

[29] Kauffman C.A. (2017). Treatment of the Midwestern endemic mycoses, blastomycosis and histoplasmosis. Curr. Fungal. Infect. Rep..

[30] Borgia S.M., Fuller J.D., Sarabia A., El-Helou P. (2006). Cerebral blastomycosis: A case series incorporating voriconazole in the treatment regimen. Med. Mycol..

[31] Bakleh M., Aksamit A.J., Tleyjeh I.M., Marshall W.F. (2005). Successful treatment of cerebral blastomycosis with voriconazole. Clin. Infect. Dis..

[32] Freifeld A., Proia L.A., Andes D., Baddour L.M., Blair J., Spellberg B., Arnold S., Lentnek A., Wheat L.J. (2009). Voriconazole use for endemic fungal infections. Antimicrob. Agents Chemother..

[33] Ta M., Flowers S.A., Rogers P.D. (2009). The role of voriconazole in the treatment of central nervous system blastomycosis. Ann. Pharmacother..

[34] Vindenes T., Gardiner B.J., Nierenberg N., Volpe G. (2015). Pneumonia, septic arthritis, and brain abscess in a construction worker. Clin. Infect. Dis..

[35] Anderson R.J., Stevens P.M., Miller F.H., Noskin G.A. (2005). Fever, progressive nonproductive cough, and headache. Infect. Dis. Clin. Pract..

[36] Martinez R. (2017). New trends in paracoccidioidomycosis epidemiology. J. Fungi.

[37] Restrepo A., Gonzalez A., Agudelo C.A., Kauffman C.A., Pappas P.G., Sobel J.D., Dismukes W.E. (2011). Paracoccidioidomycosis. Essentials of Clinical Mycology.

[38] Morejon K.M.L., Machado A.A., Martinez R. (2009). Paracoccidioidomycosis in patients infected with and not infected with human immunodeficiency virus: A case control study. Am. J. Trop. Med. Hyg..

[39] de Almeida J.N., Pecanha-Pietrobom P.M., Colombo A.L. (2019). Paracoccidioidomycosis in immunocompromised patients: A literature review. J. Fungi.

[40] Lima T.C., Bezerra R.O.F., de Brito Siqueira L.T., Menezes M.R., Leite C.D., Porta G., Cerri G.G. (2017). Paracoccidioidomycosis in a liver transplant recipient. Rev. Soc. Bras. Med. Trop..

[41] Covre L.C.P., Hombre P.M., Falqueto A., Peçanha P.M., Valim V. (2018). Pulmonary paracoccidioidomycosis: A case report of reactivation in a patient receiving biological therapy. Rev. Soc. Bras. Med. Trop..

[42] Almeida K.J., Barreto-Soares R.V., Campos-Sousa R.N., Campos-Sousa M.G., Bor-Seng-Shu E. (2018). Pulmonary paracoccidioidomycosis associated with the use of natalizumab in multiple sclerosis. Mult. Scler. J..

[43] de Almeida S.M., Queiroz-Telles F., Teive H.A.G., Ribeiro C.E., Werneck L.C. (2004). Central nervous system paracoccidioidomycosis: Clinical features and laboratorial findings. J. Infect..

[44] Pedroso V.S.P., Lyon A.C., Araujo S.A., Veloso J.M., Pedroso E.R., Teixeira A.L. (2012). Paracoccidioidomycosis case series with and without central nervous system involvement. Rev. Soc. Bras. Med. Trop..

[45] Elias J., dos Santos A.C., Carlotti C.G., Colli B.O., Canheu A., Matias C., Furlanetti L., Martinez R., Takayanagui O.M., Sakamoto A.C. (2005). Central nervous system paracoccidioidomycosis: Diagnosis and treatment. Surg. Neurol..

[46] Reis F., Collier P.P., Souza T.F., Lopes G.P., Bronzatto E., Silva Junior N.A., Pereira R.M., Appenzeller S. (2013). Neuroparacoccidioidomycosis (NPCM): Magnetic resonance imaging (MRI) findings. Mycopathologia.

[47] de Camargo Z.P. (2008). Serology of paracoccidioidomycosis. Mycopathologia.

[48] da Silva S.H.M., Colombo A.L., Blotta M.H., Queiroz-Telles F., Lopes J.D., de Camargo Z.P. (2005). Diagnosis of neuroparacoccidioidomycosis by detection of circulating antigen and antibody in cerebrospinal fluid. J. Clin. Microbiol..

[49] Wheat J., Wheat H., Connolly P., Kleiman M., Supparatpinyo K., Nelson K., Bradsher R., Restrepo A. (1997). Cross-reactivity in *Histoplasma capsulatum* variety *capsulatum* antigen assays of urine samples from patients with endemic mycoses. Clin. Infect. Dis..

[50] Shikanai-Yasuda M.A., Mendez R.P., Colombo A.L., Queiroz-Telles F., Kono A.S.G., Paniago A.M.M., Nathan A., Valle A.C.F.D., Bagagli E., Benard G. (2017). Brazilian guidelines for the clinical management of paracoccidioidomycosis. Rev. Soc. Bras. Med. Trop..

[51] Queiroz-Telles F., Goldani L.Z., Schlamm H.T., Goodrich J.M., Espinel-Ingroff A., Shikanai-Yasuda M.A. (2007). An open-label comparative pilot study of oral voriconazole and itraconazole for long-term treatment of paracoccidioidomycosis. Clin. Infect. Dis..

[52] Nelson K.E., Supparatpinyo K., Vanittanakom N., Kauffman C.A., Pappas P.G., Sobel J.D., Dismukes W.E. (2011). Penicilliosis. Essentials of Clinical Mycology.

[53] Le T., Chi N.H., Cuc N.T.K., Manh Sieu T.P., Shikuma C.M., Farrar J., Day J.N. (2010). AIDS-associated *Penicillium marneffei* infection of the central nervous system. Clin. Infect. Dis..

[54] Duong T.A. (1996). Infection due to *Penicillium marneffei*: An emerging pathogen: Review of 155 reported cases. Clin. Infect. Dis..

[55] Vanittanakom N., Cooper C.R., Fisher M.C., Sirisanthana T. (2006). *Penicillium marneffei* infection and recent advances in the epidemiology and molecular biology aspects. Clin. Microbiol. Rev..

[56] Kawila R., Chaiwarith R., Supparatpinyo K. (2013). Clinical and laboratory characteristics of penicilliosis marneffei among patients with and without HIV infection in northern Thailand: A retrospective study. BMC Infect. Dis..

[57] Stathakis A., Lim K.P., Boan P., Lavender M., Wrobel J., Musk M., Heath C.H. (2015). *Penicillium marneffei* infection in a lung transplant recipient. Transpl. Infect. Dis..

[58] Chan J.F.W., Chan T.S.Y., Gill H., Lam F.Y., Trendell-Smith N.J., Sridhar S., Tse H., Lau S.K., Hung I.F., Yuen K.Y. (2015). Disseminated infections with *Talaromyces marneffei* in non-AIDS patients given monoclonal antibodies against CD20 and kinase inhibitors. Emerg. Infect. Dis..

[59] Wang P., Chen Y., Xu H., Ding L., Wu Z., Xu Z., Wang K. (2017). Acute disseminated *Talaromyces marneffei* in an immunocompetent patient. Mycopathologia.

[60] Ye F., Luo Q., Zhou Y., Xie J., Zeng Q., Chen G., Su D., Chen R. (2015). Disseminated penicilliosis marneffei in immunocompetent patients: A report of two cases. Indian J. Med. Microbiol..

[61] Subsai K., Kanoksri S., Siwaporn C., Helen L. (2004). Neurological complications in AIDS patients: The 1-year retrospective study in Chiang Mai University, Thailand. Eur. J. Neurol..

[62] Huang Y.T., Hung C.C., Hsueg P.R. (2007). *Aspergillus* galactomannan antigenemia in *Penicilliosis marneffei*. AIDS.

[63] Le T., Kinh N.V., Cuc N.T.K., Tung N.L.N., Lam N.T., Thuy P.T.T., Cuong D.D., Phuc P.T.H., Vinh V.H., Hanh D.T.H. (2017). A trial of itraconazole or amphotericin B for HiV-associated talaromycosis. N. Engl. J. Med..

[64] Marimon R., Gene J., Cano J., Trilles L., Dos Santos Lazéra M., Guarro J. (2006). Molecular phylogeny of *Sporothrix schenckii*. J. Clin. Microbiol..

[65] de Lima Barros M.B., de Almeida Paes R., Schubach A.O. (2011). *Sporothrix schenckii* and sporotrichosis. Clin. Microbiol. Rev..

[66] Barros M.B.L., Schubach A.O., do Valle A.C.F., Gutierrez Galhardo M.C., Conceição-Silva F., Schubach T.M., Reis R.S., Wanke B., Marzochi K.B., Conceição M.J. (2004). Cat-transmitted sporotrichosis epidemic in Rio de Janeiro, Brazil: Description of a series of cases. Clin. Infect. Dis..

[67] Montenegro H., Rodrigues A.M., Dias M.A.G., da Silva E.A., Bernardi F., de Camargo Z.P. (2014). Feline sporotrichosis due to *Sporothrix brasiliensis*: An emerging animal infection in Sao Paulo, Brazil. BMC Vet. Res..

[68] Brandolt T.M., Madrid A.M., Poester V.R., Sanchotene K.O., Basso R.P., Klafke G.B., Rodrigues M.L., Xavier M.O. (2019). Human sporotrichosis: A zoonotic outbreak in southern Brazil, 2012–2017. Med. Mycol..

[69] Lipstein-Kresch E., Isenberg H.D., Singer C., Cooke O., Greenwald R.A. (1985). Disseminated *Sporothrix schenckii* infection with arthritis in a patient with acquired immunodeficiency syndrome. J. Rheumatol..

[70] Fitzpatrick J.E., Eubanks S. (1988). Acquired immunodeficiency syndrome presenting as disseminated cutaneous sporotrichosis. Int. J. Dermatol..

[71] Freitas D.F.S., Hoagland B.S., do Valle A.C.F., Fraga B.B., de Barros M.B., de Oliveira Schubach A., de Almeida-Paes R., Cuzzi T., Rosalino C.M., Zancopé-Oliveira R.M. (2012). Sporotrichosis in HIV-infected patients: Report of 21 cases of endemic sporotrichosis in Rio de Janeiro, Brazil. Med. Mycol..

[72] Bustamante B., Lama J.R., Mosquera C., Soto L. (2009). Sporotrichosis in human immunodeficiency virus infected Peruvian patients. Infect. Dis. Clin. Pract..

[73] Moreira J.A., Freitas D.F., Lamas C.C. (2015). The impact of sporotrichosis in HIV-infected patients. a systematic review. Infection.

[74] Queiroz-Telles F., Buccheri R., Benard G. (2019). Sporotrichosis in immunocompromised hosts. J. Fungi.

[75] Bunce P.E., Yang L., Chun S., Zhang S.X., Trinkaus M.A., Matukas L.M. (2012). Disseminated sporotrichosis in a patient with hairy cell leukemia treated with amphotericin B and posaconazole. Med. Mycol..

[76] Gottlieb G.S., Lesser C.F., Holmes K.K., Wald A. (2003). Disseminated sporotrichosis associated with treatment with immunosuppressants and tumor necrosis factor alpha antagonists. Clin. Infect. Dis..

[77] Gewehr P., Jung B., Aquino V., Manfro R.C., Spuldaro F., Rosa R.G., Goldani L.Z. (2013). Sporotrichosis in renal transplant patients. Can. J. Infect. Dis. Med. Microbiol..

[78] Bonifaz A., Tirado-Sanchez A. (2017). Cutaneous disseminated and extracutaneous sporotrichosis: Current status of a complex disease. J. Fungi.

[79] Donabedian H., O’Donnell E., Olszewski C., MacArthur R.D., Budd N. (1994). Disseminated cutaneous and meningeal sporotrichosis in an AIDS patient. Diagn. Microbiol. Infect. Dis..

[80] Klein R.C., Ivens M.S., Seabury J.H., Dascomb H.E. (1966). Meningitis due to *Sporotrichum schenckii*. Arch. Intern. Med..

[81] Shoemaker E.H., Bennett H.D., Fields W.S., Whitcomb F.C., Halpert B. (1957). Leptomeningitis due to *Sporotrichum schenckii*. AMA Arch. Pathol..

[82] Hessler C., Kauffman C.A., Chow F.C. (2016). The upside of bias: A case of chronic meningitis due to *Sporothrix schenckii* in an immunocompetent host. Neurohospitalist.

[83] Silva-Vergara M.L., Maneira F.R.Z., de Oliveira R.M., Santos C.T., Etchebehere R.M., Adad S.J. (2005). Multifocal sporotrichosis with meningeal involvement in a patient with AIDS. Med. Mycol..

[84] Gutierrez-Galhardo M.C., Silva M.T., Lima M.A., Nunes E.P., Schettini L.E., de Freitas R.F., Paes Rde A., Neves Ede S., do Valle A.C. (2010). *Sporothrix schenckii* meningitis in AIDS during immune reconstitution syndrome. J. Neurol. Neurosurg. Psychiatr..

[85] Freitas D.F.S., Lima M.A., de Almeida R., Lamas C.C., do Valle A.C., Oliveira M.M., Zancopé-Oliveira R.M., Gutierrez-Galhardo M.C. (2015). Sporotrichosis in the central nervous system caused by *Sporothrix brasiliensis*. Clin. Infect. Dis..

[86] Scott E.N., Kaufman L., Brown A.C., Muchmore H.G. (1987). Serologic studies in the diagnosis and management of meningitis due to *Sporothrix schenckii*. N. Engl. J. Med..

[87] Scott E.N., Muchmore H.G. (1989). Immunoblot analysis of antibody responses to *Sporothrix schenckii*. J. Clin. Microbiol..

[88] Rudramurthy S.M., Chakrabarti A. (2017). Sporotrichosis: Update on diagnostic techniques. Curr. Fungal Infect. Rep..

[89] Kauffman C.A., Hajjeh R., Chapman S.W. (2000). Practice guidelines for the management of patients with sporotrichosis. Clin. Infect. Dis..

[90] Fernandez-Silva F., Capilla J., Mayayo E., Guarro J. (2012). Efficacy of posaconazole in murine experimental sporotrichosis. Antimicrob. Agents Chemother..

